# O5.3. A COMPREHENSIVE NATIONWIDE STUDY OF COMORBIDITY WITHIN TREATED MENTAL DISORDERS – A DANISH REGISTER-BASED STUDY

**DOI:** 10.1093/schbul/sby015.217

**Published:** 2018-04-01

**Authors:** Oleguer Plana Ripoll, Carsten Pedersen, Esben Agerbo, Thomas Munk Laursen, Preben Bo Mortensen, John McGrath

**Affiliations:** 1 Aarhus University, National Center for Register-based Research; 2 Aarhus University; 3 University of Queensland, Queensland Brain Institute

## Abstract

**Background:**

Comorbidity has been the focus of a substantial body of research and it is acknowledged that different mental disorders tend to co-occur more frequently than expected. Many studies of mental disorder comorbidity have been published in recent decades, but these studies tend to be restricted to subsets of disorders, and are difficult to combine based on different methodologies. There is a need to examine comorbidity within mental disorders in a manner that covers a comprehensive range of mental disorders. The aim of this study was to use high-quality registers to (a) provide bidirectional pairwise estimates between the major groups of mental disorders, (b) investigate if associations changed depending on time since first diagnosis, (c) explore sex-specific patterns of comorbidity, and (d) estimate absolute risks rather than only incidence rate ratios of developing certain disorders after being diagnosed with one specific disorder. This abstract focus on results based on schizophrenia.

**Methods:**

We designed a population-based cohort study including all Danish residents between 2000 and 2016 (N = 5,940,778). Information on incident cases of mental diseases was obtained from the Danish Psychiatric Research Register, and we classified different disorders into 10 main groups: organic mental disorders (ICD10 F00-F09), substance abuse disorders (F10-F19), schizophrenia spectrum disorders (F20-F29), mood disorders (F30-F39), neurotic disorders (F40-F48), eating disorders (F50), personality disorders (F60), mental retardation (F70-F79), pervasive developmental disorders (F84) and behavioural and emotional disorders (F90-F98). We examined associations between all pairs of mental disorders. Hazard ratios (HR) were estimated using Cox Proportional Hazards models with age as time scale, and adjusting for sex, calendar time and other psychiatric comorbidity. Finally, we estimated the absolute risk of being diagnosed with other mental disorders after being diagnosed with a specific disorder.

**Results:**

All mental disorders were associated between them, with HR ranging from 1.1 to 19.5. There were 21,909 men and 20,106 women who were diagnosed with schizophrenia spectrum disorder (SSD) for the first time between 2000 and 2016. After onset of SSD, the rate of being diagnosed with substance abuse disorders was more than 4 times higher, compared to those without SSD (HR=4.4 [95%CI: 4.3–4.5]); the difference was larger within the first 6 months after being diagnosed with SSD (HR=31.8 [95%CI: 30.5–33.1]), although the rates remained higher even 15 years after the diagnosis (HR=3.2 [95%CI: 3.0–3.4]). Within the first 10 years after diagnosis of SSD, 23.8% [95%CI: 23.1–24.5] of men and 10.6% [95%CI: 10.2–11.1] of women were diagnosed for the first time with substance abuse disorders. Regarding mood disorders, the incidence rate was almost 3 times higher on individuals previously diagnosed with SSD than undiagnosed (HR=2.7 [95%CI: 2.6–2.7]). Analogous time-trends were observed, with larger differences within the first 6 months after diagnosis (HR=18.8 [95%CI: 18.1–19.6]), which diminished but remained higher 15 years later (HR=2.3 [95%CI: 2.2–2.4]). A total of 15.1% [95%CI: 14.5–15.6] of men and 20.8% [95%CI: 20.2–21.5] of women with a diagnosis of SSD were diagnosed with mood disorders within 10 years.

**Discussion:**

In this population-based comprehensive study, we observed that comorbidity is pervasive and usually bidirectional. We observed that after being diagnosed with a specific disorder, the risk of being diagnosed with an additional disorder is particularly higher in the first 6 months, but even after 15 years the risk is higher compared to undiagnosed individuals.

